# High Functionality Bio-Polyols from Tall Oil and Rigid Polyurethane Foams Formulated Solely Using Bio-Polyols

**DOI:** 10.3390/ma13081985

**Published:** 2020-04-24

**Authors:** Mikelis Kirpluks, Edgars Vanags, Arnis Abolins, Slawomir Michalowski, Anda Fridrihsone, Ugis Cabulis

**Affiliations:** 1 Polymer Laboratory, Latvian State Institute of Wood Chemistry, 27 Dzerbenes St., LV-1006 Riga, Latvia; edgars.vanags6@gmail.com (E.V.); arnisaabolins@gmail.com (A.A.); anda.fridrihsone@edi.lv (A.F.); cabulis@edi.lv (U.C.); 2 Department of Chemistry and Technology of Polymers, Cracow University of Technology, Warszawska 24, 31-155 Cracow, Poland; slawomir.michalowski@pk.edu.pl

**Keywords:** high functionality polyols, rigid polyurethane foam, tall oil, bio-based, thermal insulation

## Abstract

High-quality rigid polyurethane (PU) foam thermal insulation material has been developed solely using bio-polyols synthesized from second-generation bio-based feedstock. High functionality bio-polyols were synthesized from cellulose production side stream—tall oil fatty acids by oxirane ring-opening as well as esterification reactions with different polyfunctional alcohols, such as diethylene glycol, trimethylolpropane, triethanolamine, and diethanolamine. Four different high functionality bio-polyols were combined with bio-polyol obtained from tall oil esterification with triethanolamine to develop rigid PU foam formulations applicable as thermal insulation material. The developed formulations were optimized using response surface modeling to find optimal bio-polyol and physical blowing agent: c-pentane content. The optimized bio-based rigid PU foam formulations delivered comparable thermal insulation properties to the petro-chemical alternative.

## 1. Introduction

Today, the European Union (EU) acknowledges that bio-based materials play a key role in the transition from a fossil to a bio-based economy, and they are essential to the development of a more circular and decarbonized economy fossil fuel carbon intensity. The use of fossil sources as raw materials in the industry is not circular and exhibits high carbon intensity [1]. Yet raw materials from natural resources have virtually infinite renewability. Regarding sustainability, bio-based feedstocks are viewed as suitable for the circular economy model and as a way to reduce the carbon footprint [1,2,3].

One of the most suitable raw materials for the production of bio-based plastics are fatty acids that are found in plants in the form of triglycerides. The high appropriateness mainly comes from their chemical modification opportunities, ready availability, low toxicity, and especially their attractive cost [4,5,6]. The global production of vegetable oils amounted to 205 million metric tons (Mt) in 2019 [7]. The most commonly used vegetable oils are palm oil (74 Mt), soybean oil (57 Mt), rapeseed oil (27 Mt), and sunflower seed oil (21 Mt) [8,9].

The use of vegetable oil for industrial purposes competes with food and feed production [10,11]. However, there are plant oils that do not compete with food production; a good example is tall oil (TO) [12]. Crude tall oil (CTO) (which is about 38–53% fatty acids, 38–53% rosin acids, including 6–20% unsaponifiables and other residues [13]) is the third-largest side stream of the softwood Kraft pulping process after lignin and hemicellulose [14]. The separation of CTO components by fractional distillation allows obtaining tall oil fatty acids (TOFAs), which contain at least 97% fatty acids (mainly 48–52% oleic acid and 43–48% linoleic acid) and less than 3% other components, such as rosin acids and unsaponifiables [15]. TOFAs have a high content of unsaturation and this allows converting the carbon–carbon double bonds relatively easily to more useful functional groups [16]. One such easily introducible functional groups in unsaturated compounds are hydroxyl groups which are essential for producing polyurethanes (PUs) by polycondensation reaction between diverse hydroxyl groups containing compounds (polyols) and isocyanates [2,4,17,18,19].

PUs are a class of polymers that have been widely used to manufacture high-performance materials in a wide range of applications. The main applications of PUs are for flexible and rigid foams, coatings, adhesives, sealants, and elastomers [20,21,22]. In almost 80 years of its existence, the production growth of PUs has on average been quite constant [23]. In 2016, with aggregate production of 18 Mt, PUs ranked sixth in the annual worldwide polymer production [20,24]. Forecasts for the future of PUs are very optimistic due to the new markets opened in Eastern Europe, Asia, and South America [23].

Regarding polyols based on plant oil feedstock, they have attracted considerable interest throughout the world as bio-based alternatives for PU production from fossil-based polyols, thus showing a great perspective towards a greener and more sustainable future [20,25].

In recent years, a variety of methods to obtain polyols from plant oils have been studied, including: epoxidation and oxirane ring-opening [2,4,26,27,28,29,30,31,32,33,34,35,36,37,38]; hydroformylation and hydrogenation [2,4,16,36,37,39]; ozonolysis [2,4,16,36,37]; air oxidation [16,40]; dihydroxylation [16]; thiol-ene coupling [4,19,37,41,42,43]; transesterification and transamidation [4,20,26,27,28,37,38,44,45], and photochemical epoxidation (Schenck-ene reaction) [46,47,48]. However, nearly all of the published papers about polyol synthesis from plant oils are focused just on low or medium functionality/hydroxyl value polyol synthesis. Rigid PU foam production requires polyols with high average OH group functionality to ensure high mechanical, dimensional, and thermal stability of the material [49].

The most commonly used pathway for bio-polyol synthesis involves the epoxidation of plant oil fatty acid ester (triglyceride or alkyl) and subsequent oxirane ring-opening. The synthesis of the oxirane group containing intermediates is especially useful as the functionality of developed bio-polyol can be changed by using appropriate oxirane ring-opening reagents. For elastomer and flexible PU foam manufacturing, polyols with secondary OH groups and lower average functionality (f_n_ = 2–3) are more suited [49,50,51]. Polyols obtained from epoxy group opening with diethylene glycol (DEG) are more preferable for rigid PU foam production as they deliver higher functionality, f_n_ = 3–5. Furthermore, polyols with an even higher average functionality of f_n_ = 4–8 are necessary for the development of rigid PU foam formulations where polyols are used as crosslinking reagents [49,52,53]. Usually, such crosslinking polyols are obtained from petrochemical feedstock. The development of crosslinking polyols from bio-based feedstock would allow increasing the sustainable material content in the end product. Moreover, such polyols would be easier to up-scale to industrial production, as they would deliver additional benefits of material properties besides their valorization of bio-based feedstock.

Polyurethane is the sixth most used polymer on a global scale with an annual production of more than 18 million tons [54]. The majority of produced PU materials are flexible and rigid foams that are applied in various industries. The main application of rigid PU foam is as thermal insulation material in civil engineering and refrigerator appliances. Closed cell rigid PU foam thermal insulation applied in civil engineering usually has an apparent density of 40–60 kg/m^3^ with thermal conductivity of 20–30 mW/(m·K) [22,54,55]. Various sustainable feedstocks have been successfully used to develop rigid PU foams with low thermal conductivity wales, such as rapeseed oil [28,56], soybean oil [57], lignin [58], and recycled polyethylene terephthalate (PET) waste [53,59]. Although materials derived from sustainable feedstock have been used in the mentioned rigid PU foam formulations, the addition of petrochemical-based crosslinking reagents was required to develop a material with satisfactory properties.

The aim of this study was to develop rigid PU foam formulations with the end application as thermal insulation material using only bio-based polyols in the polyol component. In this study, a second-generation bio-based feedstock, TOFA, was used to develop four different bio-polyols with high average functionality. TOFA-based bio-polyols were obtained by a two-step process wherein the first step TOFA was epoxidized to introduce oxirane rings into the fatty acid backbone. In the next step of the TOFA-based bio-polyol synthesis, the oxirane rings were opened with different reagents. Furthermore, simultaneously with oxirane ring-opening, esterification of carboxylic groups of fatty acids was used to further increase the OH group functionality of the developed bio-polyols. The developed formulations were optimized to achieve rigid PU foams with an apparent density of 40 kg/m^3^, closed cell content >90%, low thermal conductivity values, typical for industry foaming parameters as well as maximal sustainable material content in the end material.

## 2. Materials and Methods

### 2.1. Materials

TOFAs (trade name FOR2) with a high content of fatty acids (>96%), and low content of rosin acids (1.9%) and unsaponifiables (1.8%) was ordered from Forchem Oyj (Rauma, Finland). Hydrogen peroxide, purum p.a., ≥35%; glacial acetic acid (EtOOH), puriss, ≥99.8%; 4-(dimethylamino)pyridine (DMAP), reagent plus, ≥99%; acetanhydride, puriss, ≥99%; dichloromethane, puriss p.a., ACS reagent; N,N-dimethylformamide (DMF), ACS reagent, ≥99.8%, water content ≤150 ppm; potassium hydroxide, puriss, ≥85%; potassium iodide, ACS reagent, ≥99%; tetraethylammonium bromide, reagent grade, 98%; perchloric acid, ACS reagent, 70%; anhydrous sodium sulfate, puriss; trimethylolpropane (TMP), reagent grade, 97%; diethylene glycol (DEG) puriss. p.a., ≥99.0% (GC), colorless; lithium perchlorate, ACS reagent, ≥95.0% were ordered from Sigma-Aldrich (Schnelldorf, Germany). Amberlite IR-120 H, strongly acidic, hydrogen form and sodium thiosulphate fixanals 0.1 M were ordered from Fluka (Bucharest, Romania). Diethanolamine (DEOA) 99.2% and triethanolamine (TEOA) 99.2% (Huntsman, Rotterdam, Netherlands) were used as purchased.

For the production of rigid PU foams the following materials were used as purchased: tris (1-chloro-2-propyl phosphate 99% (TCPP) as a flame retardant (Albermarle, Louvain-la-Neuve, Belgium); two tertiary amine-based catalysts Polycat^®^ 5 and PC CAT Q7-2 as well as 30 wt.% of potassium acetate in DEG (PC CAT TKA 30) (Air Products and Chemicals Inc., Halfweg, Netherlands); Niax Silicone L-6915 as a surfactant (Momentive Performance Materials Inc., Rotterdam, Germany); and c-pentane as a physical blowing agent (Sigma-Aldrich, Schnelldorf, Germany). In addition, the isocyanate (NCO) component for all PU materials Desmodur 44V20 L (Covestro, Krefeld, Germany) was used. It is a solvent-free mixture of 4,4′-diphenylmethane diisocyanate (MDI) oligomers with high average functionality of 2.8–2.9 and NCO content of 30.5–32.5 wt.%.

### 2.2. Epoxidation of Tall Oil Fatty Acids

The epoxidation of TOFAs was achieved by in-situ generated peroxyacetic acid which forms from acetic acid and hydrogen peroxide in the presence of an acidic catalyst. TOFA (140.0 g, 0.855 mol of C=C, 155 g I_2_/100 g) was poured into a 4 neck round bottom flask. Acetic acid (25.7 g, 0.427 mol) and an ion exchange resin Amberlite IR-120 H (28.0 g, 20% of TOFA weight) was added to the flask. The flask was immersed in a thermostatic water bath (preheated to 40 °C). A mechanical stirrer, thermocouple, and a reflux condenser were attached to the necks of the flask. The speed of the mechanical stirrer was set to 600 rpm and the mixture started to stir. Hydrogen peroxide 35% water solution (127.7 g, 1.282 mol) was poured into a dropping funnel and the funnel was attached to the round bottom flask. When the content of the flask reached 40 °C, hydrogen peroxide solution was added dropwise to the round bottom flask in a time interval of 30 min; meanwhile, the temperature of the reaction medium was slowly increased to 60 °C. After the complete addition of hydrogen peroxide, the reaction medium continued to stir for 6 h at constant conditions. Afterwards, the reaction mixture was poured into a separating funnel and washed 4 times with warm (T = 60 °C) water. The organic layer was dried on a rotatory vacuum evaporator to remove water residues. As a result, pomegranate red product, epoxidized tall oil fatty acids (ETOFAs), was obtained exhibiting the acid value of 142 mg KOH/g, oxirane content 2.31 mmol/g, and iodine value of 26.5 g I_2_/100 g.

### 2.3. Synthesis of Polyols

High functionality polyols were synthesized by functionalizing previously obtained ETOFAs. The oxirane ring-opening of ETOFAs and subsequent esterification with different polyfunctional alcohols, such as TMP, DEG, TEOA, and DEOA, were used to obtain bio-based polyols. The following acronyms were used for each polyol: ETOFA/TMP, ETOFA/DEG, ETOFA/TEOA, and ETOFA/DEOA.

**Figure 1:**
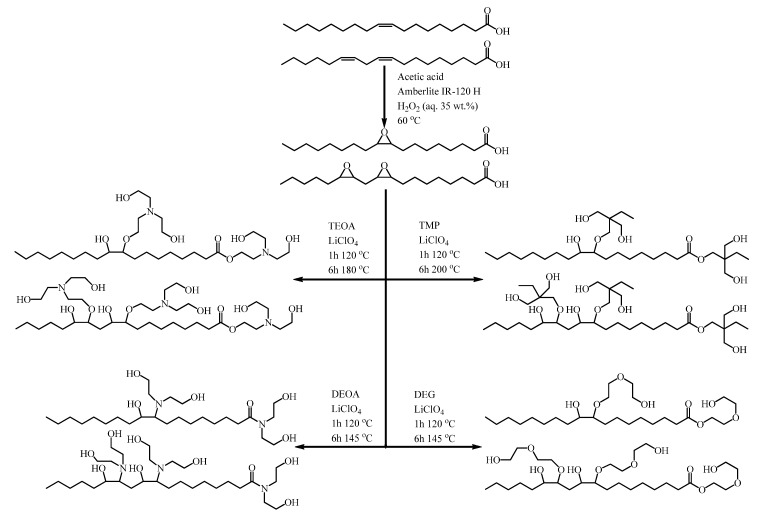
Idealized scheme of bio-polyol synthesis from TOFAs.

To obtain TOFA-based bio-polyols, first the oxirane ring-opening was carried in the four neck round bottom flask to which the multifunctional alcohol/amine (0.6776 mol; see Table 1 for corresponding mass) and lithium perchlorate LiClO_4_ as a catalyst (0.7 g; 0.5% of ETOFA mass) were added. The flask was put into an oil bath and a mechanical stirrer was inserted into the central neck. A Liebig condenser, purge gas tube, and a thermocouple were attached to the vacant necks. The mixer was set to 200 rpm and the flow of purge gas (argon) through the flask was provided, while the content of the flask was heated up to 120 °C. When the required temperature for oxirane ring-opening was reached, 140 g ETOFA (0.323 mol oxirane, 0.354 mol –COOH) was added dropwise to the flask in a time interval of 20 min. After the complete addition of ETOFA, the reaction medium continued to stir for 1 h at the temperature of 120 °C to completely open the oxirane rings. Afterwards, the synthesis temperature was increased to carry out the esterification/amidation reactions (see the corresponding temperature for each alcohol/amine used in Table 1). The stirring of the reaction medium and the argon gas flow was retained for at least five more hours until the acid value of the product decreased below 10 mg KOH/g. The idealized scheme of four high functionality bio-polyol synthesis pathways and the chemical structure of developed bio-polyols are depicted in Figure 1.

### 2.4. Characterization of Polyols and Their Precursors

The obtained bio-polyols were characterized by hydroxyl and acid values which were determined using titrimetric methods according to DIN 53240-2:2007 and DIN 53402:1990 testing standards. Epoxide content was determined according to ASTM D1652-04:2004. Iodine value was determined by the Hanus method ISO 3961:2013. The viscosity of polyols was analyzed at 25 °C using Thermo Scientific HAAKE (Medium-High Range Rotational Viscometer, Thermo Fisher Scientific, Waltham, MA, USA). Polyol density was determined by using a set of hydrometers. A graduated cylinder filled with polyol was immersed into a thermostatic bath at 20 °C. Density was measured 20 min after hydrometer immersion. The moisture content was determined by Karl Fisher titration using Denver Instrument Model 275KF automatic titrator (Denver Instrument, Bohemia, NY, USA).

Polyol structure was analyzed by Fourier transform infrared (FTIR) spectrometry data, which were obtained with a Thermo Scientific Nicolet iS50 spectrometer (Thermo Fisher Scientific, Waltham, MA, USA) at a resolution of 4 cm^−1^, 32 scans. The FTIR data were collected using attenuated total reflectance technique with ZnSe and diamond crystals. Size exclusion chromatography (SEC) from Knauer equipped with refractive index detector (Detector RI) and polystyrene/divinylbenzene matrix gel column with a measurement range up to 30,000 Da at tetrahydrofuran (THF) eluent flow of 1.0 mL/min was used to analyze the number-average molecular weight (M_n_) and number-average functionality (f_n_) of the synthesized bio-polyols. Poly(methyl methacrylate) standards with molecular mass in-between 102–18,700 Da were used to create a calibration curve. The polyols’ f_n_ was calculated based on hydroxyl values, and M_n_ as seen in Equation (1) [60].

(1) $$f_{n}=\frac{M_{n}\cdot {\mathrm{OH}}_{\mathrm{val}}}{56110}$$

where f_n_ is the number-average functionality; M_n_ is the number-average molecular weight; OH_val_ is the hydroxyl value of the polyol.

### 2.5. Rigid PU Foam Development and Formulation Optimization

The four TOFA-based bio-polyols were used for rigid PU foam development. The developed rigid PU foam formulations are depicted in Table 2. Bio-polyol with lower functionality based on tall oil (TO) esterification with TEOA (ester polyol TO_TEOA) previously developed by our research group was also used in PU foam formulations [61,62]. Varied mass ratios of the TO_TEOA with OH_val_ of 334 mg KOH/g, water content of 0.45 wt.%, viscosity of 280 mPa⋅s at 25 °C, f_n_ = 2.4 and M_n_ = 391, and newly synthesized bio-polyols were used to find optimal rigid PU foam formulations. Previous experience showed that it is necessary to add polyether type polyol into rigid PU foam formulations to enhance the bio-polyol miscibility with water. Thus, a small amount of glycerol was used. Glycerol can also be derived from renewable feedstock and thus would form the third bio-based crosslink polyol in PU formulations [63]. A combination of two blowing agents, a chemical blowing agent (distilled water) and a physical blowing agent (c-pentane) was used. The chemical blowing agent content was kept constant at 2 parts by weight (pbw) as its variation would impact the chemical composition of the rigid PU foam polymer matrix. The amount of physical blowing agent was varied between 0–15 pbw and its influence on the apparent density of the developed rigid PU foam was studied. A constant amount of TCPP flame retardant (8 wt.%) of PU foam mass was selected for all developed rigid PU foam formulations. TCPP is commonly used in rigid PU foams that are applied in civil engineering. Lastly, all rigid PU foam formulations had a set isocyanate index of 150. The catalyst package was adjusted for each of the TOFA-based bio-polyol series to ensure good PU foam properties.

Furthermore, sustainable material content, as well as the green carbon content of the developed materials were calculated. The sustainable material content was expressed as the total mass of renewable feedstock in PU formulation divided by the total mass of PU formulation; the value was multiplied by a factor of 100, and result expressed in percent (%). The sustainable material content of each synthesized bio-polyol is depicted in Table 1, whereas tall oil content in TO_TEOA polyol was 60%. The green carbon content was expressed as the total mass of green carbon in renewable feedstock divided by the total amount of carbons in PU formulation; the value was multiplied by a factor of 100 and expressed in percent (%). For bio-polyols, the idealized structure was assumed in the calculation. The green carbon content was slightly higher than sustainable material content because the non-renewable raw materials contain many non-carbon elements, such as nitrogen, phosphorous, and chlorine.

The substances listed in Table 2 (green polyols, catalyst, blowing agent, surfactant, and flame retardant) were used to prepare the polyol component of the rigid PU foams by stirring them for 1 min with a mechanical stirrer at 2000 rpm. Afterwards, the polyol component was conditioned at room temperature for at least 2 h to release mixed in air. Rigid PU foams were obtained by mixing polyol and isocyanate (pMDI) components with a mechanical stirrer at 2000 rpm for 15 s and the reacting PU foam mass was poured into an open-top mold.

As seen in Table 2, a few of the used reagents are depicted as a range between certain values. This depicts the experimental matrix of the rigid PU foam formulation optimization. Three factors of the rigid PU foam formulations were changed: the content of the newly developed TOFA-based bio-polyols, the content of the low functionality TO_TEOA polyol, and the content of the physical blowing agent c-pentane in part by weight ratios of 0–95, 95–0, and 0–15, respectively. The experiment matrix of the changed factors is depicted in Table 3. Following c-pentane content for each new TOFA-based bio-polyol and TO_TEOA polyol ratios were tested at 0, 3, 6, 9, 12, and 15 pbw. In total, 36 rigid PU foam formulations were tested for each of the four different TOFA-based bio-polyols amounting to 144 tested formulations.

The altered factor influence on rigid PU foam properties was optimized using response surface modeling (RSM) which was done with the help of Design Expert software (Version V12.0.7.0, Stat-Ease, Inc. Minneapolis, MN, USA). The following responses were investigated which were tested according to respective testing standards: apparent density ISO 845:2006, closed cell content ISO 4590:2016, and technological rigid PU foam foaming parameters (start time, string time, tack-free time, and rise time—cup test methodology). A linear or polynomic model was fitted for each response that optimized rigid PU foam formulation in targeted constraints and delivered a desirability surface. The most optimal rigid PU foam formulation and a few others in the area of highest desirability were selected to obtain larger scale rigid PU foam samples to test the thermal conductivity and compression strength. The thermal conductivity was measured according to ISO 8301:1991 at an average temperature of 10 °C (cold plate: 0 °C and hot plate: +20 °C, sample dimensions: 200 × 200 × 30 mm). The compression strength of rigid PU foams was tested perpendicular and parallel to the foaming direction using Zwick/Roell Z100 testing machine (Zwick Roell Group, Ulm, Germany), standard EN ISO 844:2014, maximum load-cell capacity 1 kN, the deformation rate: 10%/min) for cylinder specimens with diameter and height of ~20 mm. Six specimens were analyzed for each formulation. The optimization of rigid PU foam formulation resulted in several systems that could be applied as thermal insulation material in the construction industry.

## 3. Results and Discussion

### 3.1. Characterization of Synthesized TOFA-Based Bio-Polyols

Two subsequent processes carried out high functionality TOFA-based bio-polyol synthesis from ETOFAs. The first step of high functionality TOFA-based bio-polyol synthesis was TOFA epoxidation using in-situ formed peroxyacetic acid; this step was described in a previous study by Vanags et al. [64]. The epoxidized TOFA was used as a raw material for the subsequent bio-polyol synthesis. The second step was ETOFA oxirane ring-opening and esterification/amidation of fatty acid carboxyl groups with various molecular sub-units, such as TMP, DEG, TEOA, and DEOA. As epoxy ring-opening catalyst LiClO_4_ was used, but the esterification/amidation reaction was insured by the increase of the reaction medium temperature as described in Table 1. The extent of the novelty consisted in the principle that the carboxylic groups of TOFAs were not blocked before TOFA epoxidation. The described synthesis approach complied with the principles of Green chemistry and simplifyied the overall process by circumventing an additional synthesis step. Unfortunately, this resulted in undesired side reactions of oxirane ring-opening with carboxylic groups of TOFAs, which formed dimers, trimers, and further higher molecular mass oligomers.

**Figure 2:**
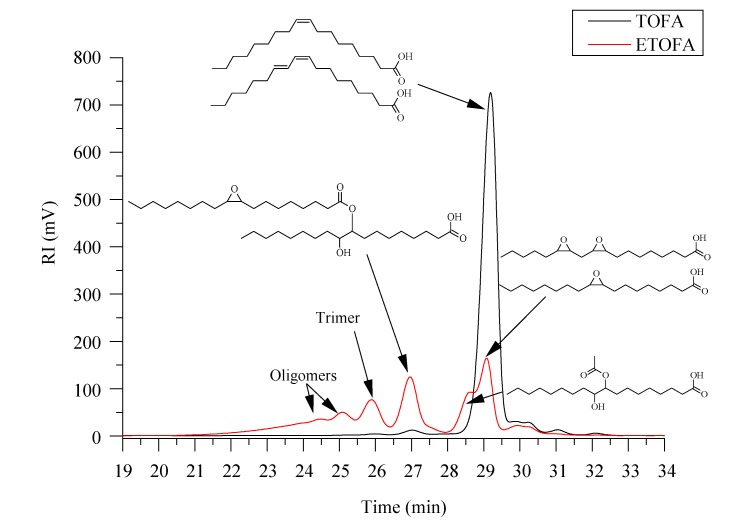
Size exclusion chromatography (SEC) graph of neat TOFAs and ETOFAs used for the bio-polyol synthesis.

**Figure 3:**
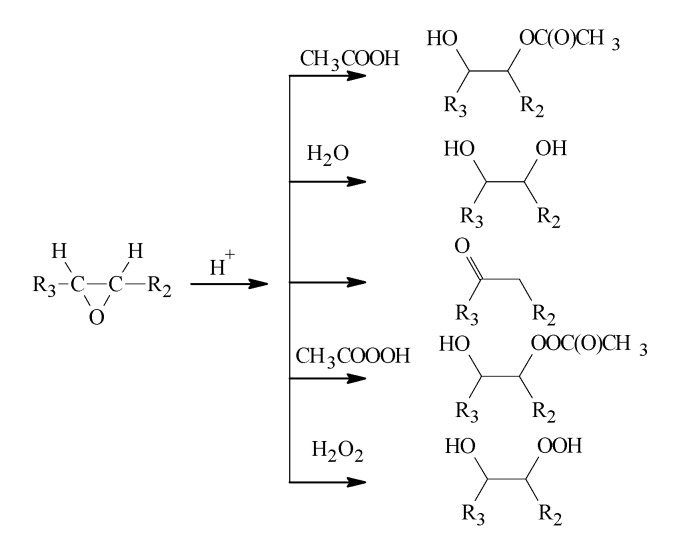
The possible side-reactions of epoxide rings in acidic medium.

The chemical structures of ETOFAs and possible side reaction products, as well as their average molecular mass, are depicted in Figure 2. Side reaction formation is clearly seen in SEC analysis when neat TOFAs are compared with ETOFAs (Figure 2.). In addition, other possible side reactions may occur during the epoxidation process (Figure 3). The formation of oligomerization products is undesired because it reduces the overall content of oxirane groups along with increasing the viscosity of ETOFAs and subsequent bio-polyols. The selectivity of the TOFA epoxidation was estimated to be only 54% (please see our previous publication [64]). Nevertheless, the obtained ETOFAs can be considered as a suitable raw material for bio-polyol synthesis as they exhibit relatively high oxirane oxygen content of 2.21 mmol/g or 3.54% of oxirane oxygen.

The main characteristics of synthesized TOFA-based bio-polyols, such as hydroxyl value, viscosity, acid value, moisture content, density, number average functionality, average molar mass, and polydispersity, are summarized in Table 4. The synthesized TOFA bio-polyols have a relatively high OH value that is necessary and typical for polyols to be used for rigid PU foam development. The high viscosity of bio-polyols could be problematic for further upscale of the technology as it will limit their application due to limitations of the processing equipment. However, the viscosity of synthesized polyols is comparable with Lupranol^®^3422, a sorbitol-based polyol with f_n_ = 6, produced by BASF which has a viscosity of 22,750 mPa·s [65]. Synthesized high functionality bio-polyols can be compared to Lupranol^®^3422 and other polyols that are obtained from propyl oxidation of sorbitol, such as R-4720 from Repsol, Voranol™ RN 482 from Dow, and Rokopol^®^ RF55 from PCC Rokita, as the number average functionality of synthesized polyols is quite high at f_n_ = 5.0–9.3. The high f_n_ of the synthesized bio-polyols makes them suitable as crosslinking reagents in PU material development.

**Figure 4:**
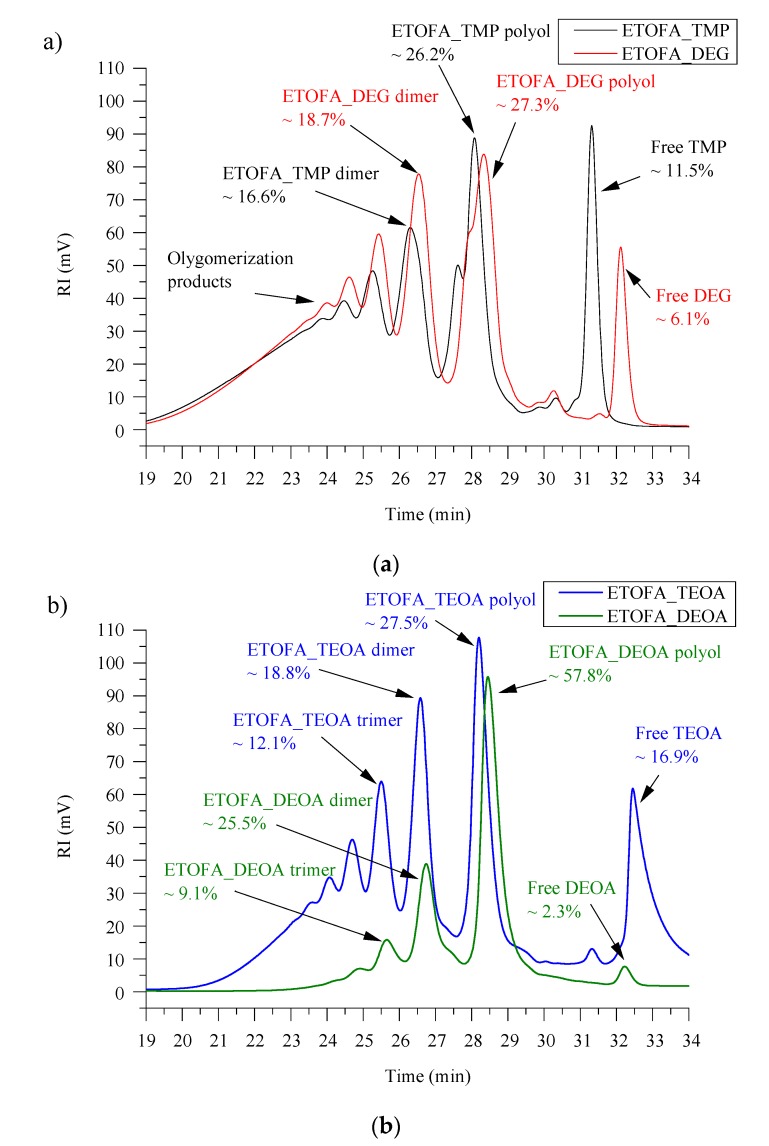
SEC analysis of TOFA bio-polyols. (**a**) ETOFA_TMP and ETOFA_DEG; (**b**) ETOFA_TEOA and ETOFA_DEOA.

All four TOFA bio-polyols are a mixture of several oligomerization products as seen from SEC analysis depicted in Figure 4. The largest peak area for the polyol structures is depicted in Figure 1. Each consecutive peak is for the dimerization, trimerization, and oligomerization products that are obtained due to TOFA dimerization during epoxidation as well as oligomerization during the oxirane ring-opening reaction. The presence of several different molecular mass products together with the branched structure of the TOFA-based bio-polyols is the reason for the relatively high viscosity. Nevertheless, this does not hinder their application in the rigid PU foam development. It must be mentioned that ETOFA_TME, ETOFA_DEG, and ETOFA_TEOA polyols contain a significant number of unreacted reagents used for oxirane ring-opening and esterification of TOFA carboxylic groups: 11.5%, 6.1%, and 16.9%, respectively. In the case of ETOFA_DEOA polyol, the amount of free DEOA is only ~2.3%, indicating that DEOA is significantly more selective towards ETOFA oxirane ring-opening and carboxylic group amidation. This is also confirmed by a much lower amount of oligomerization products and lower polydispersity (p_d_ = 1.28) of ETOFA_DEOA polyol. The significantly higher viscosity of ETOFA_DEOA polyol is explained by the hydrogen bonding of the polar amide groups present in the bio-polyol structure.

### 3.2. Characterization of the Chemical Structure of Synthesized Bio-Polyols Using FTIR

**Figure 5:**
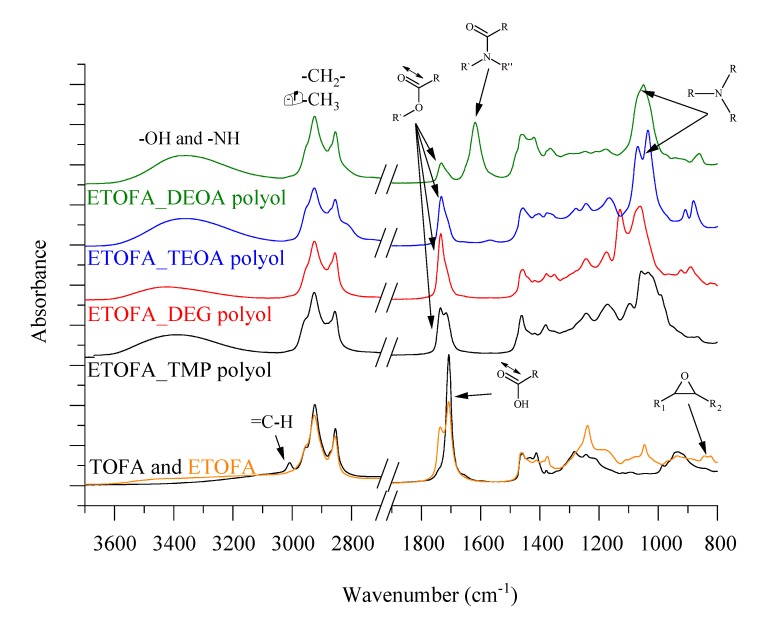
Fourier transform infrared (FTIR) spectra of TOFAs, ETOFAs, and the resulting four TOFA-based bio-polyols.

The conversion of functional groups during each step of polyol synthesis was studied by FTIR spectroscopy. The FTIR spectra of TOFA, ETOFA, and resulting bio-polyols are presented in Figure 5. The broad absorption peak between 3600–3125 cm^−1^ for all spectra of synthesized polyols is characteristic for stretching vibrations of OH and NH groups. The intensity of OH group absorption band correlates with titrimetric OH value results presented in Table 4 where ETOFA_DEG polyol has the lowest height peak. TOFA, ETOFA, and the obtained bio-polyols had typical peaks of –CH_2_ symmetric and asymmetric stretching at ~2930 and 2860 cm^−1^.

TOFA-based bio-polyol synthesis can be followed by the change in oxirane ring vibration peak and by the change of C=O stretching position. The oxirane ring vibration peak at 823 cm^−1^ of ETOFA disappeared in TOFA bio-polyol FTIR spectra which confirms the conversion towards the desired reaction. Moreover, the oxirane ring-opening was confirmed by the titrimetric analysis method where the oxirane oxygen content in all four TOFA-based bio-polyols was below 0.01 mmol/g. The side reactions during TOFA epoxidation depicted in Figure 2 are confirmed by the development of a shoulder peak of C=O ester stretching at 1736 cm^−1^ for ETOFA intermediate product. The carboxylic group C=O stretching vibration at 1707 cm^−1^ of TOFAs disappeared for ETOFA_TMP, ETOFA_DEG, and ETOFA_TEOA polyols as it shifted towards C=O stretching vibration of esters at ~1733 cm^−1^.

ETOFA_TEOA and ETOFA_DEOA polyols have a significant difference in FTIR spectra when compared with ETOFA_TMP and ETOFA_DEG polyols. A tertiary amine group was identified at ~1036 cm^−1^ that could provide autocatalytic properties of the developed bio-polyols. Furthermore, the amide structure of ETOFA_DEOA was confirmed by C=O stretching vibration peak shift to 1625 cm^−1^. ETOFA_DEOA polyol contains a mixture of DEOA esters as well as amides similar to structures reported by Stirna et al. [66].

### 3.3. Development of ETOFA_TMP Polyol Based Rigid PU Foam

All four developed high functionality TOFA-based bio-polyols have the potential to be applied as crosslinking reagents in the rigid PU foam material formulations due to higher than average OH group functionality at a relatively low molecular weight. Three factors were optimized for each PU foam series, the content of TOFA-based bio-polyol, the content of TO_TEOA polyol, and the content of physical c-pentane blowing agent. Experimental factors were changed according to the matrix described in Table 3. Six different responses were evaluated, such as apparent density of rigid PU foam, closed cell content, sustainable material content in rigid PU foams, foaming start time, PU foam string time, and PU foam tack-free time. The emphasis was put to obtain rigid PU foams with an apparent density of 40 ± 5 kg/m^3^, closed cell content above >90%, and maximum sustainable material content in rigid PU foams.

**Figure 6:**
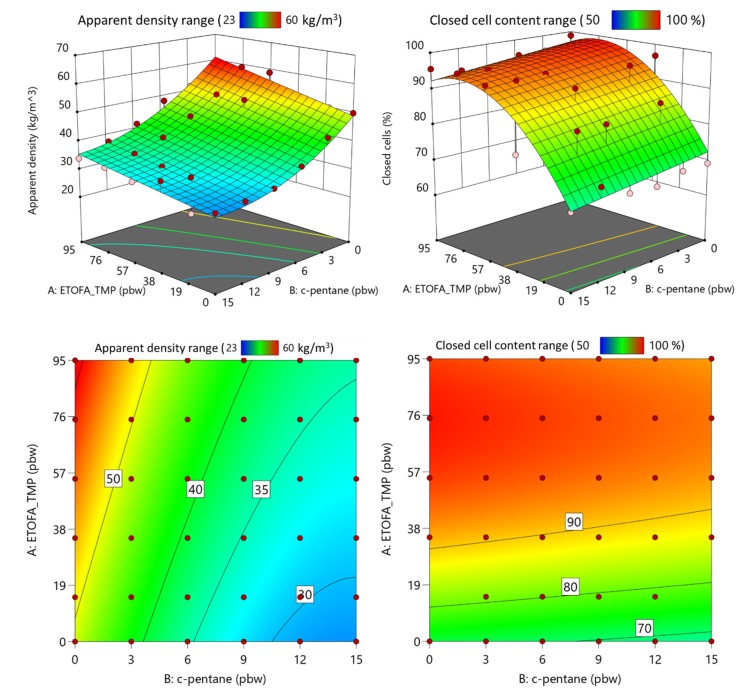
Response surfaces of ETOFA_TMP polyol and c-pentane influence on the developed rigid PU foam apparent density and closed cell content.

The data were analyzed employing multiple regression techniques to develop RSM. The altered factor influence on the selected responses was approximated using the linear or second-order polynomial model at the 95% confidence level. The developed models were further optimized to remove non-significant terms of the model (*p*-value less than 0.05). TOFA-based bio-polyol and TO_TEOA polyol content in rigid PU foam formulations are mutually dependent in the selected experimental matrix, thus only the content of one polyol can be used as a factor in the model development. The obtained response surface of ETOFA_TMP polyol and c-pentane influence on rigid PU foam apparent density and closed cell content is presented in Figure 6 where determined values are depicted as the red points above the response surface and pink points below the response surface. The determined coefficients for the rigid PU foam parameter response surface models are summarized in Table 5. The developed models fit reasonably well in the experimental data with R^2^ values larger or close to 0.9. The developed models can be used to predict rigid PU foam foaming parameters and optimize desired properties. An example of the response surface equation for one of the parameters is described by Equation (2), where A is the content of TOFA-based bio-polyols, but B is the content of c-pentane in pbw.

Response = X_1_ + X_2_·A + X_3_·B + X_4_·A·B + X_5_·A^2^ + X_6_·B^2^ (2)

The decrease of the apparent density of ETOFA_TMP rigid PU foam series with the increase of c-pentane content is self-evident (Figure 6). Furthermore, the polyol composition also had a slight influence on the apparent density of the developed rigid PU foams. It decreased with the decrease of ETOFA_TMP polyol content. It can be explained by two aspects; first, the viscosity of ETOFA_TMP polyol is much higher than the viscosity of TO_TEOA: 77,000 and 280 mPa⋅s, respectively. The higher viscosity of ETOFA_TMP hinders the rigid PU foam bubble expansion, and thus the increase of apparent density of rigid PU foam with the increase of ETOFA_TMP polyol content. The second aspect is related to the chemical structure of both polyols. ETOFA_TMP polyol does not have tertiary amine groups in its structure (see Figure 5), whereas TO_TEOA does. Higher reactivity and autocatalytic properties of TO_TEOA polyol along with its lower viscosity decreased the apparent density of the developed rigid PU foam. Apparent density is a paramount parameter of rigid PU foam as it influences not only physical properties of the material, but also is important as an economical factor. A thermal insulation producer would be interested to use as little of the material as possible while maintaining good thermal insulation performance. The closed cell structure of rigid PU foam is also essential to reach low thermal conductivity values as they directly correlate with the blowing agent gas content locked inside rigid PU foam cells. The closed cell content was only slightly changed by different c-pentane content as depicted in Figure 6. The polyol type used in rigid PU foam formulation had the largest influence on the closed cell content of the material. Figure 6 shows that it is necessary to use high functionality polyols in rigid PU foam formulation to obtain a material with closed cell morphology. This is directly related to the chemical structure of PU polymer matrix and its crosslinking density. It was possible to obtain rigid PU foams with closed cell content above 90% with an apparent density as low as 31 kg/m^3^ when ~44 pbw of ETOFA_TMP polyol was used in rigid PU foam formulation.

**Figure 7:**
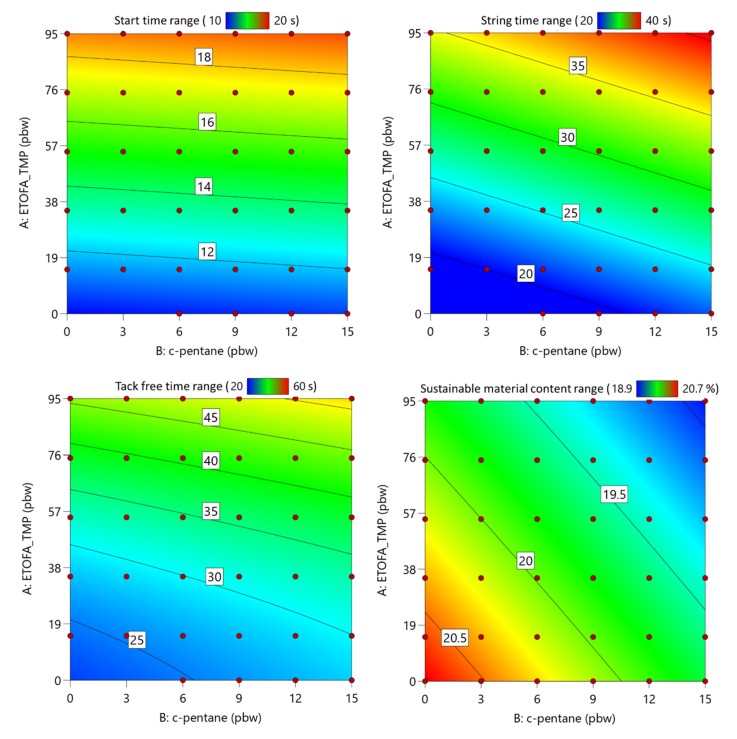
Response surfaces of ETOFA_TMP polyol and c-pentane influence on the developed rigid PU foam start time, string time, tack-free time, and sustainable material content.

Rigid PU foam foaming parameter RSM analysis is depicted in Figure 7. C-pentane content had little influence on the start time of PU foam foaming. It decreased with decreasing content of ETOFA_TMP polyol and increasing content of TO_TEOA polyol due to the autocatalytic properties of the latter. The increase of c-pentane content slightly increased PU foam string and tack-free time as the physical blowing agent cools down the PU foam mass during the foaming process, thus reducing the reactivity of the material. The sustainable material content in rigid PU foam is also depicted in Figure 7. This parameter was not measured but calculated from the mass of tall oil used in the different polyol synthesis. Sustainable material content decreased with the addition of c-pentane and ETOFA_TMP polyol.

Similar data were obtained for the other three TOFA-based bio-polyols. The response surface figures of ETOFA_DEG polyol, ETOFA_TEOA, ETOFA_DEOA, and c-pentane influence on the developed rigid PU foam apparent density, closed cell content start time, string time, tack-free time, and sustainable material content are depicted in Figures S1–S3 of the Supplementary Material, respectively. Moreover, the RSM model coefficients of ETOFA_DEG polyol, ETOFA_TEOA, ETOFA_DEOA polyol series are summarized in Tables S1–S3, respectively. The influence of the other three TOFA-based bio-polyols on rigid PU foam properties was similar to ETOFA_TMP polyol with a few exceptions. ETOFA_TEOA and ETOFA_DEOA bio-polyols did not significantly change the rigid PU foam foaming parameters, because their chemical structure contains tertiary amine groups, the same as for TO_TEOA polyol. Rigid PU foam developed using ETOFA_DEOA had decreased closed cell content of ~70% which is not satisfactory. The main issue was ETO_DEOA polyol miscibility with other components due to its high viscosity. Furthermore, ETOFA_DEOA polyol mixing with isocyanate was also problematic which resulted in very low dimensional stability of the PU foam. In the present formulations, ETOFA_DEOA polyol was not suitable for further thermal insulation material development. The obtained ETOFA_TMP, ETOFA_DEG, and ETOFA_TEOA polyol RSM surface models were used to optimize rigid PU foam formulations.

### 3.4. Optimization of Developed Rigid PU Foam Formulations

Multiple response optimization was done by using an objective function *D(X)*, called the desirability function. It is a function of the desirable range for each response (*d_i_*). Each response is described by a desirable range from one to zero, where one is the most desirable and zero is the least desirable. The simultaneous objective function is a geometric mean of all transformed responses described by Equation (3), where *n* is the number of responses. Furthermore, different values of importance were assigned to each response relative to the other responses. The importance (*r_i_*) was varied from least important at the value of 1, to the most important at the value of 5. If the response or factors fall outside their desirability range, the overall function becomes zero [67].

(3) $$D={\left(d_{1}{}^{r_{1}}\cdot d_{2}{}^{r_{2}}\cdot \ldots \cdot d_{n}{}^{r_{n}}\right)}^{\frac{1}{\sum^{}r_{i}}}={\left(\overset{n}{\underset{i=1}{\prod }}d_{i}{}^{r_{i}}\right)}^{\frac{1}{\sum^{}r_{i}}}$$

For simultaneous optimization, target values were selected for each response which are summarized in Table 6. Furthermore, the different importance level values were assigned for each target value. Rigid PU foams with an apparent density of 40 ± 5 kg/m^3^ were targeted as such material is most commonly used in civil engineering applications. The closed cell content above 90% and the maximal sustainable material content was prioritized. Lastly, rigid PU foam foaming parameters in the typical range for hand-mixed formulations were selected.

**Figure 8:**
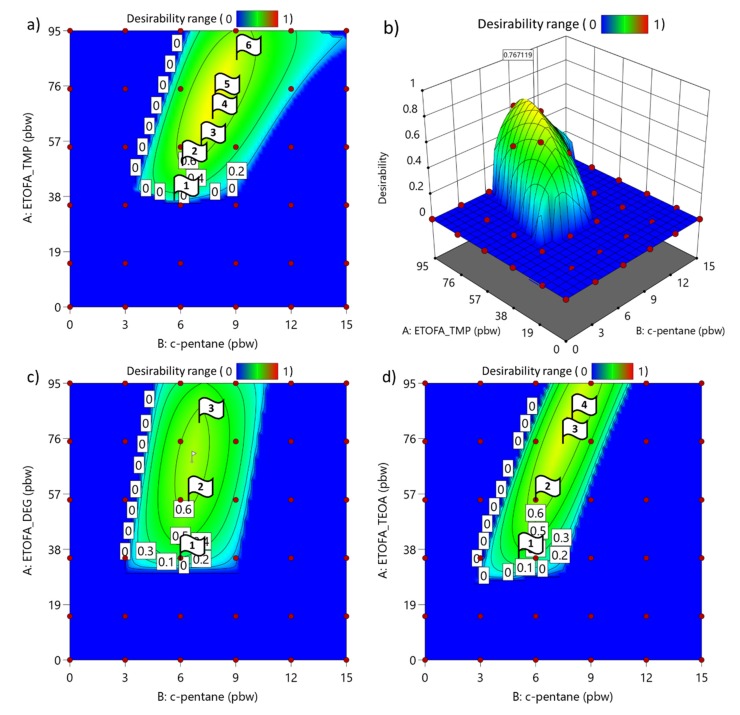
Desirability response of the rigid PU foam optimization: (**a**) ETOFA_TMP polyol series; (**b**) ETOFA_TMP polyol series plotted as three-dimensional (3D) surface; (**c**) ETOFA_DEG series; and (**d**) ETOFA_TEOA series.

The obtained desirability response from Equation (3) can be plotted as a surface in-between changed content of TOFA bio-polyol and c-pentane. For ETOFA_DEOA polyol series the desirability analysis was not done, because it was not possible to obtain rigid PU foams with closed cell content above 90% due to high viscosity of the polyol and miscibility problems with other components. The obtained desirability responses are depicted in Figure 8. The highest value of desirability and few additional formulations in the area of high desirability response were selected to produce larger rigid PU foam samples for further testing. The selected formulations with corresponding flag numbers from Figure 8 and their desirability response are presented in Table 7. The other components of rigid PU foam formulation were selected the same as described in Table 2. The isocyanate index for all rigid PU foams was 150.

### 3.5. Development of Rigid PU Foam Thermal Insulation Using Optimized Formulations

**Figure 9:**
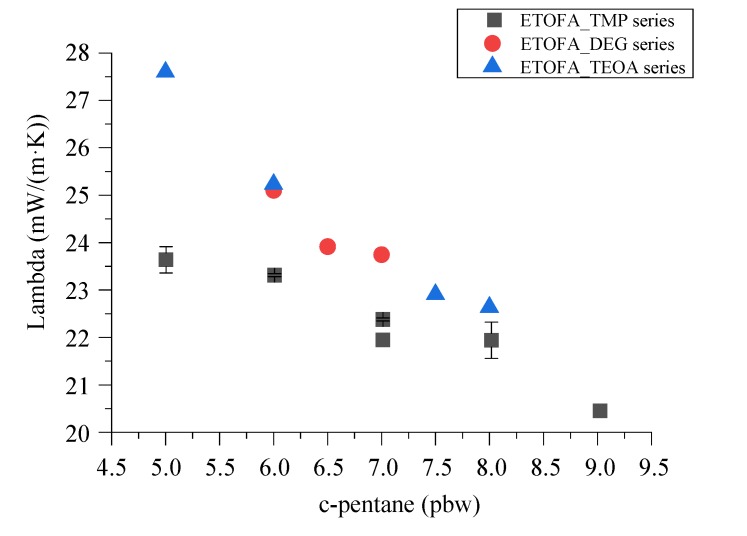
Thermal conductivity of the optimized rigid PU foam formulations.

Rigid PU foam formulations described in Table 7 were used to obtain larger samples of rigid PU foam material to measure the thermal conductivity. The apparent density between all samples was in the range of 37.7–40.5 kg/m^3^ which closely fits with the predictions made by the developed model from Table 5, and Tables S1 and S2. Furthermore, the closed cell content for all of the larger rigid PU foam samples was as expected, above 90%. The main goal of the study was to obtain good quality thermal insulation material with a low value of thermal conductivity coefficient λ, which was achieved (Figure 9). The obtained λ values were in direct correlation with the content of the physical blowing agent c-pentane used in the foam material. The λ values were as low as 20.5–23.7 mW/(m·K) which is considered acceptable for industrial application. Unfortunately, rigid PU foam formulation from ETOFA_TEOA polyol at 35 pbw and c-pentane content at 5 pbw had a quite high initial λ value of 27.6 mW/(m·K), which is not satisfactory. A higher λ value directly correlates with comparably low c-pentane content and with the chemical structure of used polyol. ETOFA_TEOA polyol content of 35 pbw is deemed to be too low to obtain a highly crosslinked PU polymer matrix which is required to retain a blowing agent inside of the closed cells. The crosslink density influence on rigid PU foam thermal conductivity can also be judged from the λ values of ETOFA_TMP polyol series. They were the lowest when compared to the other two TOFA bio-polyols which correlates with the high average OH group functionality of the polyol.

**Figure 10:**
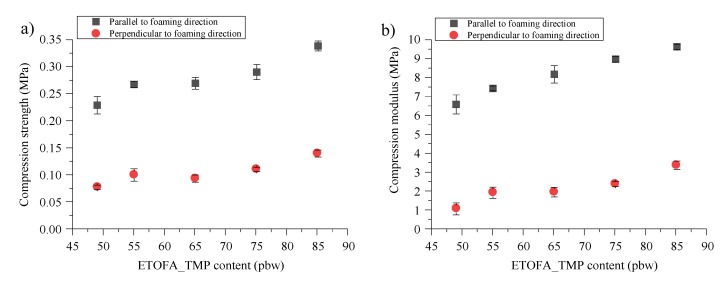
Compression properties of ETOFA_TMP polyol based rigid PU foams: (**a**) compression strength (**b**) compression modulus.

The compression strength was measured for ETO_TMP polyol based rigid PU foams because they showed the best thermal conductivity performance in comparison with other TOFA-based bio-polyols. This was done to identify the most commonly used characteristics of developed thermal insulation material and to compare them with industrially used reference material. The compression strength and compression modulus data for ETOFA_TMP based rigid PU foams were measured parallel and perpendicular to the foaming direction, as depicted in Figure 10. The developed ETOFA_TMP polyol based rigid PU formulations had especially good agreement with predicted values of the apparent density. Average values of apparent density between all tested compression strength materials were 39.1 ± 1.3 kg/m^3^ which is very close to the desired value of 40 kg/m^3^. Rigid PU foam compression strength and compression modulus had an almost linear correlation with the content of ETOFA_TMP polyol in the formulation. This is explained by the increase in the crosslink density of the PU polymer matrix due to high ETOFA_TMP polyol functionality. The different content of c-pentane had little effect on the rigid PU foam mechanical properties, although it is expected that it could decrease the mechanical properties because c-pentane acts as a plasticizer.

The common properties of the developed rigid PU foam formulations were compared to typical commercially used spray, applied rigid PU foam thermal insulation material from BASF (Table 8) and a commercial mixture of polyester polyol from Purinova. Furthermore, the developed rigid PU foams were compared to other rigid PU foams developed using sustainable feedstocks, such as PPWC-0T—tall oil based polyol; RO/PET_150—polyol from recycled PET and rapeseed oil, and F_0_—formulation based on sucrose/glycerin initiated polyether polyols. The chosen rigid PU foam formulations from literature data were selected, because they had similar apparent density and isocyanate index to developed rigid PU foam. The initial thermal conductivity of the developed rigid PU foam formulations was similar or better than for compared materials. It must be mentioned that the comparison material was foamed using hydrofluorocarbon (HFO) blowing agents. HFO blowing agents usually deliver superior thermal conductivity properties because c-pentane tends to leak from rigid PU foam over time. The long term stability of thermal conductivity for developed materials must be tested for further upscale of the technology. The high functionality of ETOFA_TMP polyol obtained rigid PU foams with much higher compression properties at the same apparent density when compared to reference foams. The developed rigid PU foam had sustainable material content of 19.4% which can be considered relatively high as more than half of PU material mass is derived from polyisocyanate. The high viscosity of ETOFA_TMP polyol could be problematic for further upscale. Unfortunately, the viscosity of the polyol component was not measured, but it is expected that it will be lower than for neat ETOFA_TMP polyol.

## 4. Conclusions

Tall oil fatty acids were used as a second-generation feedstock to obtain four different high functionality bio-polyols. Bio-polyols were synthesized without solvent and harmful byproducts did not form during the synthesis. The obtained TOFA-based bio-polyols were characterized with a high number average functionality of 5.0–9.3 and relatively low molecular mass of 569–1215 g/mol. It was demonstrated that it is possible to obtain good quality rigid PU foam thermal insulation using only bio-polyols in the polyol component of the material. Synthesized high functionality bio-polyols are required to obtain rigid PU foams with closed cell structure. The chemical structure of developed bio-polyols had a direct influence on the foaming parameter of the developed polyurethane formulations as bio-polyols containing tertiary amine groups delivered autocatalytic properties. The obtained bio-polyols were used to develop rigid PU foam thermal insulation with an apparent density of ~40 kg/m^3^ and thermal conductivity as low as 20.5–23.7 mW/(m·K). Best quality rigid PU foams for civil engineering application were obtained using ETO_TMP with lower thermal conductivity values when compared to other TOFA-based bio-polyols. A response surface model that predicts the apparent density of rigid polyurethane foams when the content of bio-polyols and blowing agent is changed was developed. The developed rigid polyurethane foams had similar thermal conductivity as commercially used alternatives but ~46% higher compression strength properties.

## Figures and Tables

**Table 1:** Multifunctional alcohols/amines used in the bio-polyol syntheses.

Multifunctional Alcohol for Oxirane Ring-Opening and Esterification of TOFA ^1^	Mass, g	Esterification/Amidation Temperature, °C	TOFA Content in Bio-Polyol, %
TMP ^1^	90.9	200	62.2
DEG ^1^	71.9	200	67.9
TEOA ^1^	101.1	180	59.5
DEOA ^1^	71.2	145	68.1

^1^ TOFA, tall oil fatty acid; TMP, trimethylolpropane; DEG, diethylene glycol; TEOA, triethanolamine; DEOA, diethanolamine.

**Table 2:** Rigid polyurethane (PU) foam formulations, green carbon, and sustainable material content in rigid PU foams.

Components		Different Reagents, pbw			
		ETOFA_TMP	ETOFA_DEG	ETOFA_TEOA	ETOFA_DEOA
Polyols	ETOFA_TMP *	95–0	−	−	−
	ETOFA_DEG *	−	95–0	−	−
	ETOFA_TEOA *	−	−	95–0	−
	ETOFA_DEOA *	−	−	−	95–0
	TO_TEOA *	0–95	0–95	0–95	0–95
	Glycerol *	5	5	5	5
Blowing agents	Water *	2.0	2.0	2.0	2.0
	c-pentane	0–15	0–15	0–15	0–15
Catalysts	Amine Catalysts	4.0	2.5	4.0	4.0
	PC CAT TKA 30	1.5	1.5	1.5	1.5
Surfactant	L6915	2.5	2.5	2.5	2.5
Flame retardant	TCPP	8 wt.%	8 wt.%	8 wt.%	8 wt.%
Isocyanate	pMDI	155–183	138–155	163–209	156–197
Green carbon content in %		18.1–26.4	24.4–26.5	16.7–26.5	18.7–26.2
Sustainable material content in %		15.8–23.2	20.8–23.7	15.1–23.5	16.3–23.1

* Feedstock partially or fully based on renewables. ETOFA, epoxidized tall oil fatty acid.

**Table 3:** Experimental matrix of the changed factors for rigid PU foam formulation optimization.

Changed Factor	Weight Ratios of Changed Factors, pbw					
New TOFA-based bio-polyols	95	75	55	35	15	0
TO_TEOA	0	20	40	60	80	95
c-pentane	0–15	0–15	0–15	0–15	0–15	0–15

**Table 4:** The main characteristics of TOFA-based bio-polyols.

Polyol	OH val., mg KOH/g	Viscosity (25 °C), mPa⋅s	Acid val., mg KOH/g	Moisture, %	Density (20 °C), g/cm^3^	f_n_	M_n_	p_d_
ETOFA/TMP	390 ± 15	77,000 ± 1000	7 ± 2	<0.1	1.056	9.3	1264	1.59
ETOFA/DEG	260 ± 10	1060 ± 40	5 ± 2	<0.1	1.039	5.8	1215	1.53
ETOFA/TEOA	500 ± 15	7400 ± 100	3 ± 2	<0.5	1.047	7.9	888	1.71
ETOFA/DEOA	480 ± 15	104,000 ± 2000	2 ± 2	<0.2	1.070	5.0	569	1.28

**Table 5:** The coefficients of the response surface model for rigid PU foams developed using ETOFA_TMP polyols and the R^2^ values of the model.

Response	X_1_	X_2_	X_3_	X_4_	X_5_	X_6_	R^2^
Apparent density	+48.98633	+0.129410	−2.82320	−0.003643	0	+0.096614	0.9665
Closed cell content	+72.43408	+0.713941	−0.312739	0	−0.004800	0	0.9004
Start time	+10.06081	+0.090974	+0.036797	0	0	0	0.8611
String time	+15.89392	+0.197703	+0.390999	0	0	0	0.8743
Tack-free time	+22.47031	+0.090724	+0.383783	0	+0.001611	0	0.9200

**Table 6:** Targeted values of developed rigid PU foams for material optimization.

Optimized Responses	Targeted Value	Importance Level
Apparent free rise density	40 ± 5 kg/m^3^	5
Closed cell content	MAX (>90%)	5
Sustainable material content in the rigid PU foam	MAX	3
Start time	~10–20 s	0–1
String time	~20–40 s	0–1
Tack-free time	~30–60 s	0–1

**Table 7:** Rigid PU foam formulations selected from desirability response surfaces.

**Flag Nr**	**ETOFA_TMP Polyol Content, pbw**	**c-pentane Content, pbw**	**TO_TEOA Polyol Content, pbw**	**Desirability**
1	35	5	60	0.20
2	49	6	44	0.60
3	55	7	40	0.68
4	65	7	30	0.74
5	75	8	20	0.77
6	85	9	10	0.71
**Flag Nr**	**ETOFA_DEG Polyol Content, pbw**	**c-pentane Content, pbw**	**TO_TEOA Polyol Content, pbw**	**Desirability**
1	35	6	60	0.40
2	55	6.5	40	0.63
3	84	7	11	0.62
**Flag Nr**	**ETOFA_TEOA Polyol Content, pbw**	**c-pentane Content, pbw**	**TO_TEOA Polyol Content, pbw**	**Desirability**
1	35	5	60	0.46
2	55	6	40	0.65
3	75	7.5	20	0.71
4	83	8	12	0.71

**Table 8:** ETOFA_TMP bio-polyol based rigid PU foam comparison with other materials.

Characteristics	ETOFA_TMP—85 c-pentane—9	PPWS-0T ^1^	RO/PET_150 ^2^	Rigid PU Foam F_0_^3^	Elastospray 1622/6 BASF System ^4^	PU-0 ^5^
Apparent density, kg/m^3^	37.7	38.8	39.1	37.4	37.0	39.0
Closed cell content, %	94	n.a.	99	86	95	88
Thermal conductivity, mW/(m·K)	20.5	27.4	20.7	25.5	20.5	n.a.
Compression strength, MPa	0.32	0.22	0.35	0.21	0.22	0.25
Compression modulus, MPa	9.0	4.9	7.0	5.1	n.a.	6.4
Sustainable mat. content, %	19.4	n.a.	~12.5	n.a.	n.a.	n.a.
Average cell size, μm	240 ± 84	840 ± 50	n.a.	530 ± 170	n.a.	390 ± 9
Isocyanate index	150	150	150	110	n.a.	160

^1^ Data were taken from A. Kairyte et al. [68]; ^2^ Data were taken from A. Ivdre et al. [59]; ^3^ Data were taken from A. Septevani et al. [69]; ^4^ Data were taken from Elastospray 1622/6 BASF technical datasheet [70]; ^5^ Data were taken from S. Czlonka et al. [71].

## Supplementary Materials

The following are available online at https://www.mdpi.com/1996-1944/13/8/1985/s1, Figure S1: The influence of ETOFA_DEG polyol and c-pentane on the developed rigid PU foam apparent density, closed cell content start time, string time, tack free time and sustainable material content, Figure S2: The influence of ETOFA_TEOA polyol and c-pentane on the developed rigid PU foam apparent density, closed cell content start time, string time, tack-free time and sustainable material content, Figure S3: The influence of ETOFA_DEOA polyol and c-pentane on the developed rigid PU foam apparent density, closed cell content start time, string time, tack-free time and sustainable material content, Table S1: The coefficients of the response surface model for rigid PU foams developed using ETOFA_DEG polyol and the R^2^ values of the model, Table S2: The coefficients of the response surface model for rigid PU foams developed using ETOFA_TEOA polyol and the R^2^ values of the model., Table S3: The coefficients of the response surface model for rigid PU foams developed using ETOFA_DEOA polyol and the R^2^ values of the model.
