# Supplementary material for: High Functionality Bio-Polyols from Tall Oil and Rigid Polyurethane Foams Formulated Solely Using Bio-Polyols
Source: Materials (Basel). 2020 Apr 24;13(8):1985. doi: 10.3390/ma13081985 (PMC7215456; doi:10.3390/ma13081985)
Supplement: Supplementary file 1 [file materials-13-01985-s001.pdf]

materials

MDPI

Article

High Functionality Bio-Polyols from Tall Oil and Rigid Polyurethane Foams Formulated Solely Using Bio-Polyols

Mikelis Kirpluks [1,*], Edgars Vanags [1], Arnis Abolins [1], Slawomir Michalowski [2], Anda Fridrihsone [1] and Ugis Cabulis [1]

[1] Polymer Laboratory, Latvian State Institute of Wood Chemistry, 27 Dzerbenes St., LV-1006 Riga, Latvia; edgars.vanags6@gmail.com (E.V.); arnisaabolins@gmail.com (A.A.); anda.fridrihsone@edi.lv (A.F.); cabulis@edi.lv (U.C.)

[2] Department of Chemistry and Technology of Polymers, Cracow University of Technology, Warszawska 24, 31-155 Cracow, Poland; slawomir.michalowski@pk.edu.pl

* Correspondence: mkirpluks@gmail.com;

Received: 27 March 2020; Accepted: 22 April 2020; Published: 24 April 2020

Supplementary Materials:

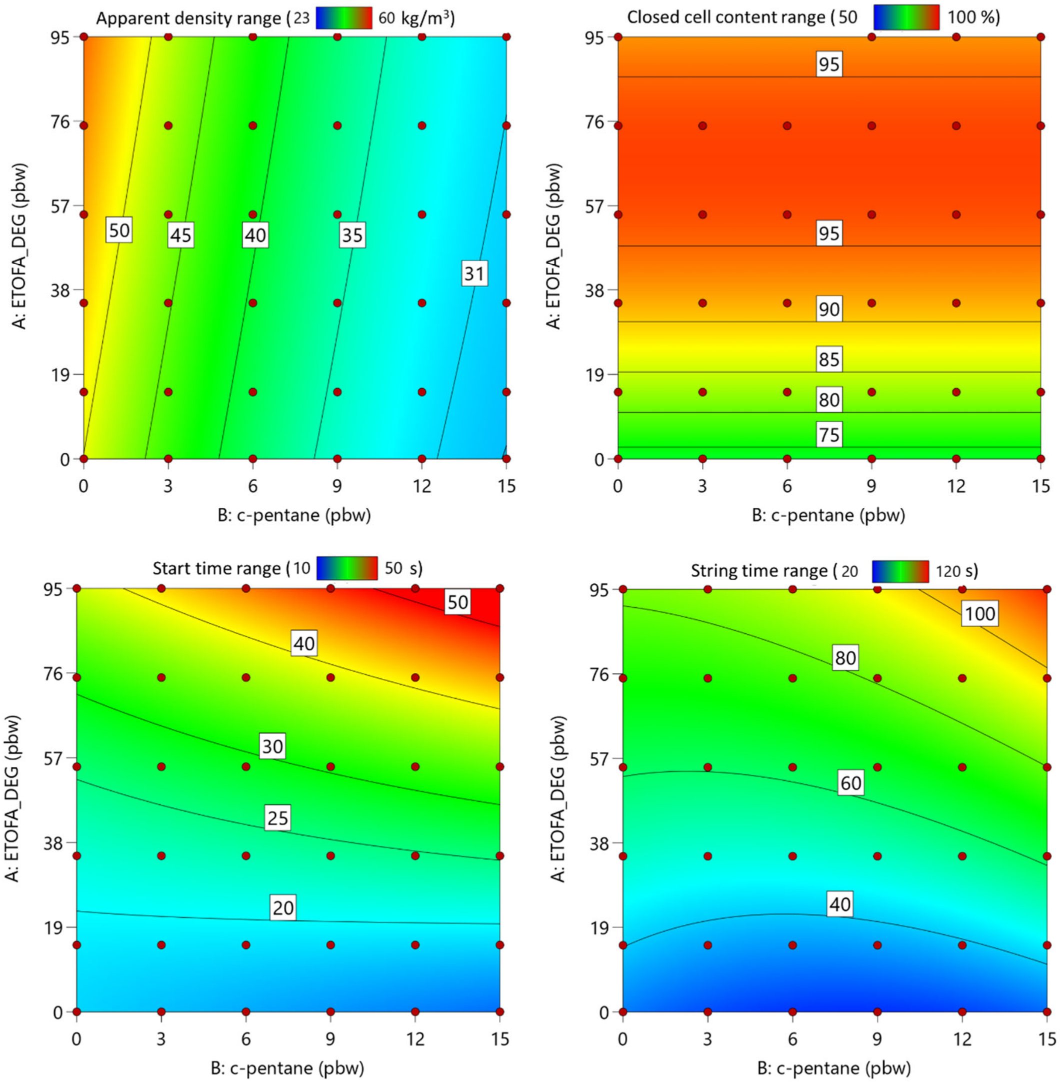

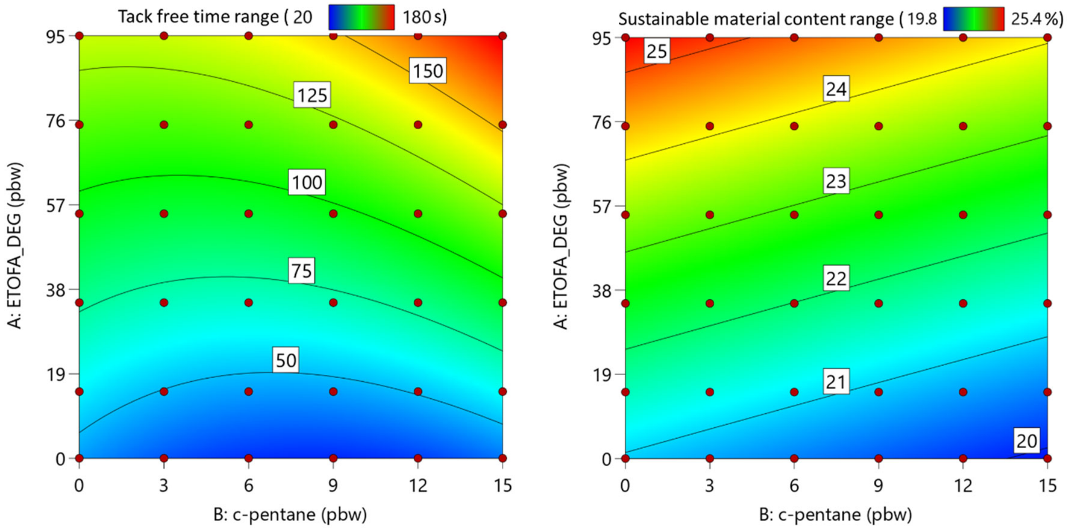

Figure S1. The influence of ETOFA_DEG polyol and c-pentane on the developed rigid PU foam apparent density, closed cell content start time, string time, tack free time and sustainable material content.

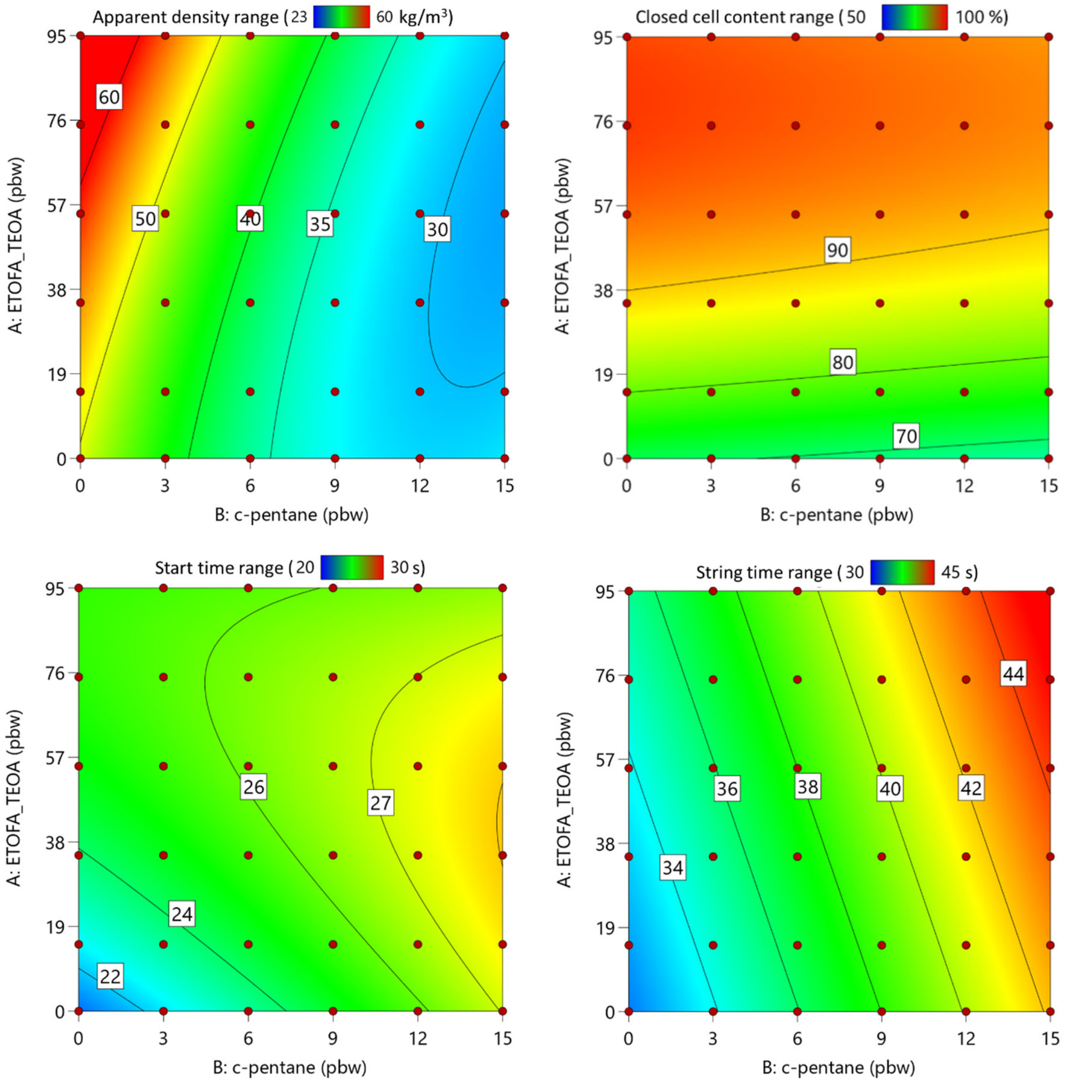

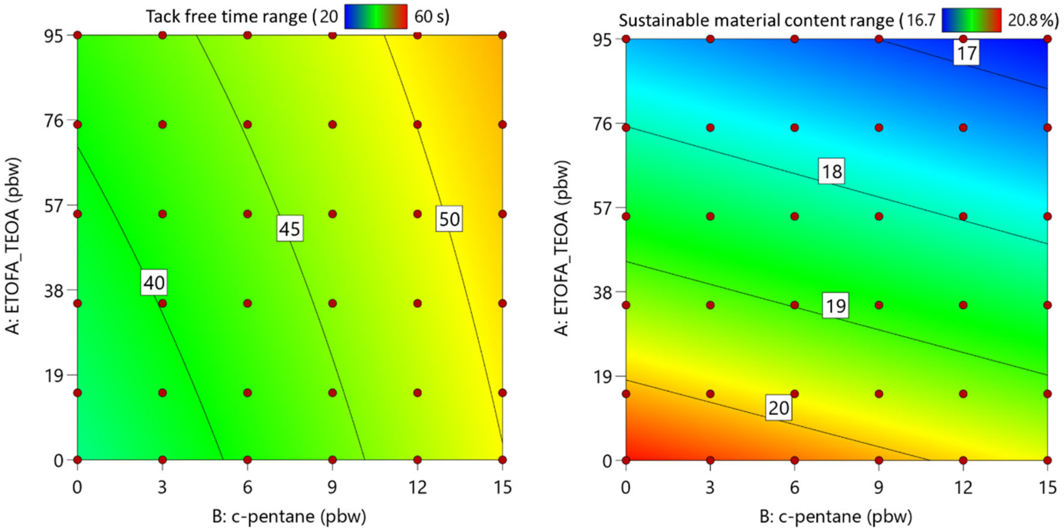

Figure S2. The influence of ETOFA_TEOA polyol and c-pentane on the developed rigid PU foam apparent density, closed cell content start time, string time, tack-free time and sustainable material content.

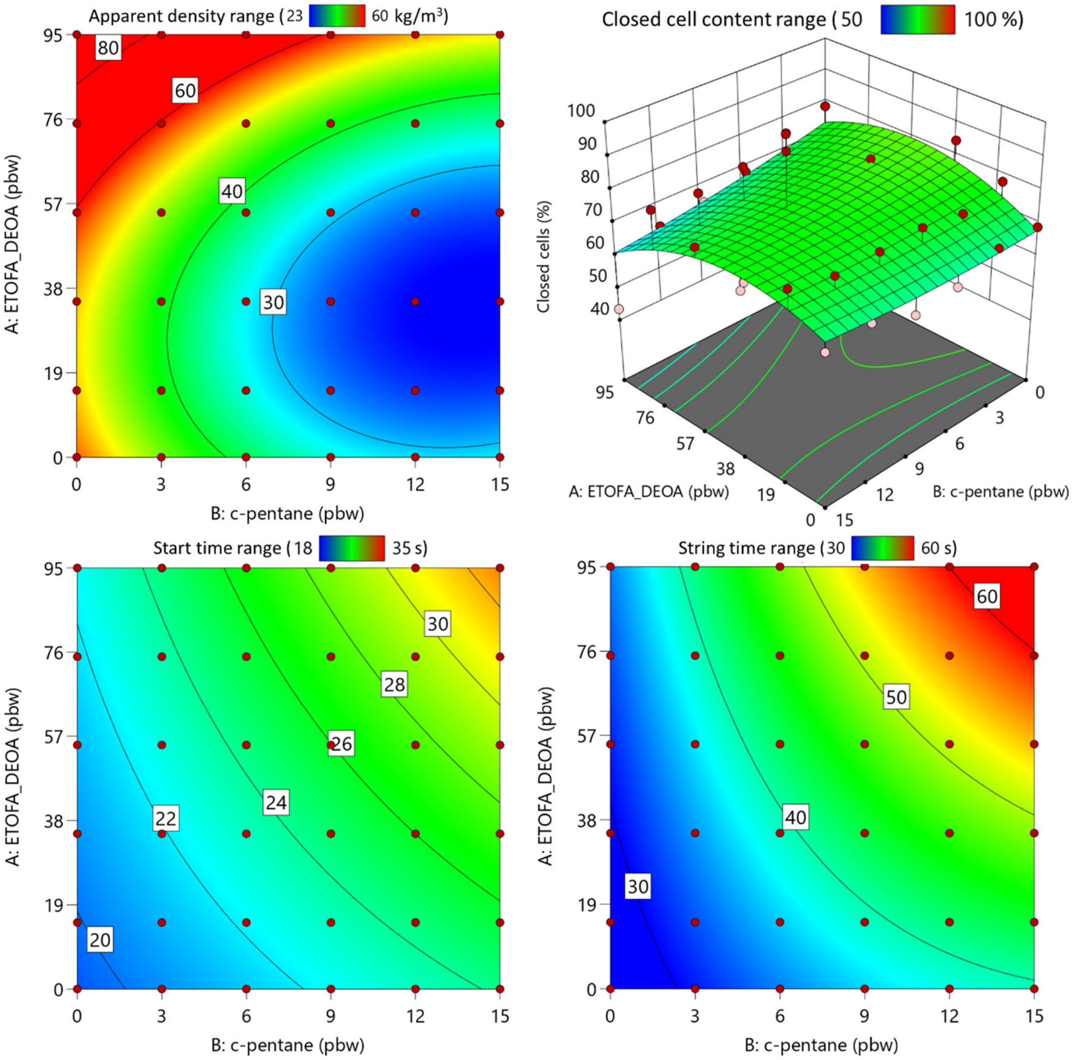

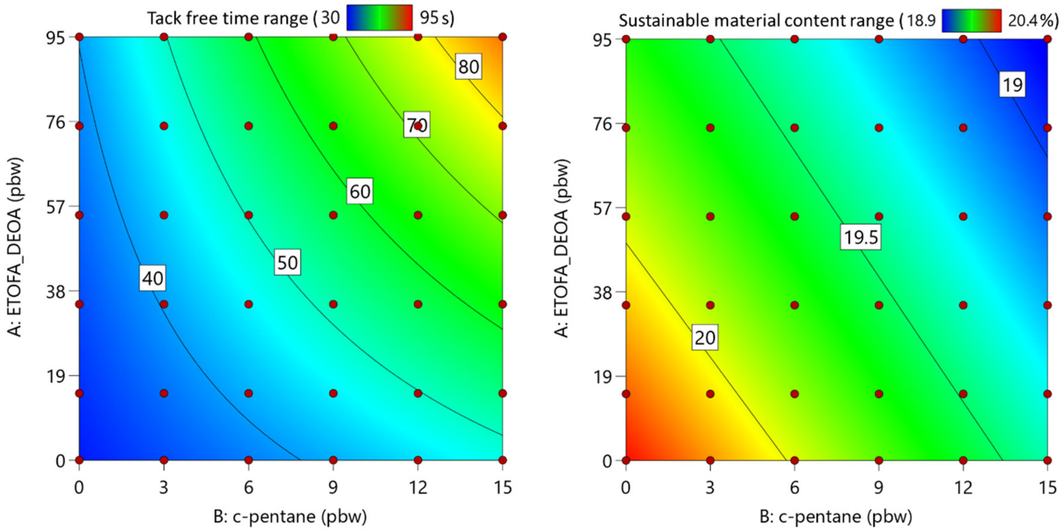

Figure S3. The influence of ETOFA_DEOA polyol and c-pentane on the developed rigid PU foam apparent density, closed cell content start time, string time, tack-free time and sustainable material content.

Table S1. The coefficients of the response surface model for rigid PU foams developed using ETOFA_DEG polyol and the R^2 values of the model.

Response	X_1	X_2	X_3	X_4	X_5	X_6	R^2
Apparent density	+49.92072	+0.065856	−2.41701	−0.003495	0	+0.072417	0.9708
Closed cell content	+73.17936	+0.708681	−0.05289	0	0	0	0.8034
Start time	+18.41085	+0.027359	−0.270835	+0.014740	+0.001898	0	0.9052
String time	+32.39577	+0.521625	−1.90821	+0.025266	0	+0.118056	0.9143
Tack free time	+44.68807	+0.920962	−4.41700	+0.039968	0	+0.266204	0.9154

Table S2. The coefficients of the response surface model for rigid PU foams developed using ETOFA_TEOA polyol and the R^2 values of the model.

Response	X_1	X_2	X_3	X_4	X_5	X_6	R^2
Apparent density	+49.56239	+0.118197	−2.94824	−0.013988	+0.000843	+0.116085	0.9736
Closed cell content	+71.22987	+0.647049	−0.265714	0	−0.004001	0	0.9360
Start time	+21.08180	+0.100972	+0.397109	−0.003504	−0.000579	0	0.7551
String time	+31.80652	+0.037295	+0.690476	0	0	0	0.8712
Tack free time	+34.84132	+0.073592	+1.00192	−0.002605	0	0	0.9150

Table S3. The coefficients of the response surface model for rigid PU foams developed using ETOFA_DEOA polyol and the R^2 values of the model.

Response	X_1	X_2	X_3	X_4	X_5	X_6	R^2
Apparent density	+56.45725	-0.381397	−3.90823	−0.010869	+0.007899	+0.150556	0.9373
Closed cell content	+68.16171	+0.433562	−0.215256	−0.009499	−0.003988	+0.020440	0.2132
Start time	+19.45946	+0.030841	0.316446	+0.003970	0	0	0.7823
String time	+27.01791	+0.072077	+1.33598	+0.013163	0	−0.033730	0.9520
Tack free time	+31.59988	+0.090808	+1.07074	+0.021937	0	0	0.9349

© 2020 by the authors. Submitted for possible open access publication under the terms and conditions of the Creative Commons Attribution (CC BY) license (http://creativecommons.org/licenses/by/4.0/).
